# Transitions in cognitive test scores over 5 and 10 years in elderly people: Evidence for a model of age-related deficit accumulation

**DOI:** 10.1186/1471-2318-8-3

**Published:** 2008-02-18

**Authors:** Arnold Mitnitski, Kenneth Rockwood

**Affiliations:** 1 Department of Medicine & Geriatric Medicine Research Unit, Dalhousie University & QEII Health Sciences Centre, Capital District Health Authority, Halifax, Nova Scotia, Canada; 2 Department of Mathematics and Statistics, Dalhousie University, Halifax, Nova Scotia, Canada

## Abstract

**Background:**

On average, health worsens with age, but many people have periods of improvement. A stochastic model provides an excellent description of how such changes occur. Given that cognition also changes with age, we wondered whether the same model might also describe the accumulation of errors in cognitive test scores in community-dwelling older adults.

**Methods:**

In this prospective cohort study, 8954 older people (aged 65+ at baseline) from the Canadian Study of Health and Aging were followed for 10 years. Cognitive status was defined by the number of errors on the 100-point Modified Min-Mental State Examination. The error count was chosen to parallel the deficit count in the general model of aging, which is based on deficit accumulation. As with the deficit count, a Markov chain transition model was employed, with 4 parameters.

**Results:**

On average, the chance of making errors increased linearly with the number of errors present at each time interval. Changes in cognitive states were described with high accuracy (R^2 ^= 0.96) by a modified Poisson distribution, using four parameters: the background chance of accumulating additional errors, the chance of incurring more or fewer errors, given the existing number, and the corresponding background and incremental chances of dying.

**Conclusion:**

The change in the number of errors in a cognitive test corresponded to a general model that also summarizes age-related changes in deficits. The model accounts for both improvement and deterioration and appears to represent a clinically relevant means of quantifying how various aspects of health status change with age.

## Background

As people age, most notice that their cognitive function changes, usually for the worse. In general, aspects of recent memory are less efficient, especially the divided attention required for working memory [1,2]. How to understand those changes is controversial. Whether the changes are inevitable, and somehow benign, or the beginning of a decline that will be seen as pathologic is not clear [3].

Descriptions of processes of cognitive ageing abound, and several reports evaluate longitudinal change using a variety of models [4-10] but comparatively fewer accounts quantify how cognition changes with age, i.e. predict the numeric degree to which scores change. We have modeled other age-related changes and demonstrated that a few parameters can be estimated to accurately describe how deficits accumulate with age [11,12]. Inasmuch as the prevalence of all forms of cognitive impairment increases with age [13], as do deficits in general, we wondered whether the patterns of general deficit accumulation might also obtain with decrements in cognition. Here, we investigated how community-dwelling elderly people accumulate errors in cognitive performance over 5–10 years.

## Methods

### The cognitive measure

The Modified Mini-Mental state Examination [14] is a 100-point scale that was based on Folstein's Mini-Mental State Examination (MMSE) [15]. The 3MS adds to the MMSE by including tasks of animal naming, similarities, date and place of birth and a second recall task, and by providing more rigorous scoring. This expansion of the testing of cognitive and of precision, have generally resulted in better psychometric properties of the 3MS [15,16].

### Cognitive states defined by 3MS errors

For these analyses, we propose that variation in 3MS scores can describe cognitive states. Analogous to the 'deficits' counted in the frailty index to define varying states of health, we defined cognitive states in relation to cognitive deficits. A deficit can be considered in relation to the 3MS. Recent work by our group suggests that a 2-point change on the 3MS over 5 years is clinically detectable, [17] in that it represents an effect size (Cohen's *d*) of 0.5, considered to be a 'medium' effect size, and readily visible in the course of normal experience. [18] Thus, we consider that the "0" state of cognitive errors, i.e. the lowest level of cognitive impairment detected by the test, to be defined as a score of 100 or 99, i.e. of having no or 1 error. The "1" state represents 3MS scores of 98 or 97, i.e. 2 or 3 errors and so on.

If cognitive states can be defined by the number of 3MS errors/cognitive deficits present, then it might be that cognitive deficits accumulate as general health deficits do. If so, the techniques that can be employed to analyze health in relation to deficits accumulated in a frailty index should be able to be applied to analyze cognition in relation to the accumulation of cognitive deficits. Specifically, it could be that the distribution of errors in cognition, and the chance of accumulating fewer errors, or more errors, or maintaining the same number of errors, or dying might conform to patterns seen with the frailty index, i.e. cognitive deficits might show the same patterns as general health deficits.

### The sample

The Canadian Study of Health and Aging (CSHA) is a representative cohort study, the chief aim of which was to estimate epidemiological aspects of dementia and other age-related problems [19]. The methods are described in detail elsewhere [20]. Briefly, community-dwelling participants were screened using the 3MS, from which a sub-sample was selected for more detailed clinical and neuropsychometric evaluation. Here, we consider the community-dwelling sample (n = 9,008 at baseline – CSHA-1) and those participants (n = 8,954) who were able to complete the 3MS. Of these, complete data are available for 5,586 survivors for the second wave (CSHA-2, conducted in 1995–1996) and 3,211 for the third wave (CSHA-3, conducted in 2000–2001). Complete mortality follow-up is available, so we know that 1,821 people died in the 60-month interval between CSHA-1 and CSHA-2.

### Analysis

Earlier we have shown that the probabilities of transitions between two states *n *and *k *(*P*_*nk*_) can be represented by a modified Poisson distribution [11,12,21]

(1) $$P_{nk}=\frac{{\bar{k}}_{n}^{k}}{k!}\exp(-{\bar{k}}_{n})\cdot (1-P_{nd}),$$

where ${\bar{k}}_{n}$ is positive parameter that depends on the current state *n *and linearly increases with *n*: ${\bar{k}}_{n}={\bar{k}}_{0}+\beta_{1}n.$. We must also take into account the so-called 'absorbing state' of death. *P*_*nd *_is probability to die for any given *n *state at baseline; it increases exponentially:

(2) *P*_*nd *_= *P*_*0d *_exp(*β*_2_*n*)

(*P*_*nd *_≤ 1). Of the four parameters, two can be considered as the background components: $\bar{k_{0}}$, *P*_*0d*_, and *β*_1_, and *β*_2 _(*the increments*), $\beta_{1}={\bar{k}}_{n+1}-{\bar{k}}_{n}$, and *β*_2 _is the slope of the log death probability can be estimated from observational data. $\bar{k_{0}}$ is the mean number of cognitive errors in transition for the people who had zero errors at baseline (at the zero state) and *P*_*0d *_is the probability of 5-year mortality for the people at the zero state (with zero cognitive errors at baseline). The increments show how these values change with the baseline error state number. In short, we illustrate four state-dependent parameters, in two of which ($\bar{k_{0}}$, *P*_*0d*_,) the parameter estimate refers to the 0-state, i.e. no cognitive errors. We refer to them as background parameters, in that changes here reflect the influence of insults arising at an environmental level (including the internal environment) and will be added to the changes at all other levels – note that, at the 0-state, there is no possibility for improvement. The other two parameters (*β*_1_, and *β*_2_) illustrate that the transition probabilities depend on the starting state, so that someone at error state 4, for example, will, on average, expect worse outcomes (both a greater chance for worsening and a higher chance of dying) than will someone at error states 0,1,2 or 3.

To estimate the parameters of the model and their confidence intervals, a nonlinear fitting procedure was used. The codes were written in Matlab 7.3 (Matworks Inc.). The parameters were estimated for each transition separately. Goodness of fit was evaluated using *R*^2^.

### Ethics

The CSHA protocol was approved by ethics committees at each of the 36 participating centres, and each participant (or proxy) gave written, informed consent. Permission for these secondary analyses was granted by the Research Ethics Committee of the Capital District Health Authority, Halifax, Canada.

## Results

**Figure 1:**
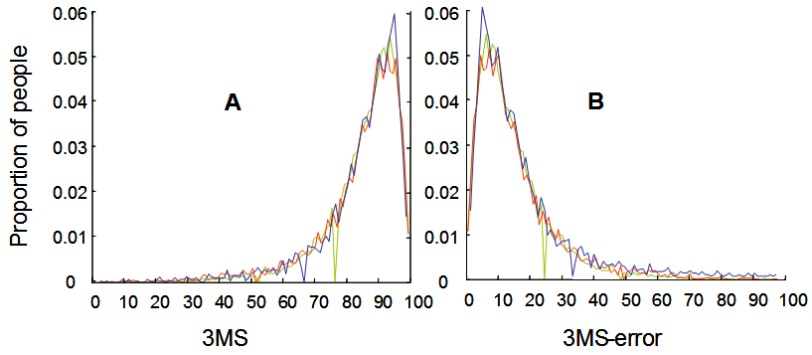
**The distribution of Modified Mini-Mental State Examination scores**. The distribution of Modified Mini-Mental State Examination scores (Panel A) and 3MS-errors (Panel B) at baseline (CSHA-1, red), in 5 years, CSHA-2 (blue) and in 10 years, CSHA-3 (green).

**Figure 2:**
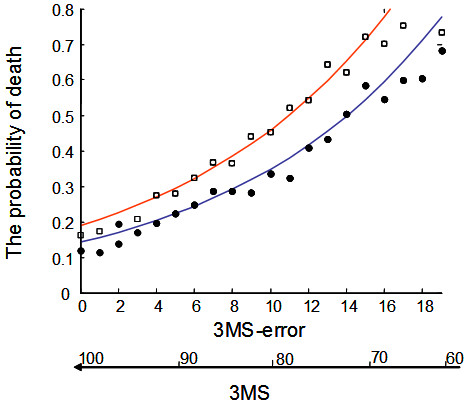
**Death probability as a function of the number of errors on the Modified Mini-Mental State Examination Score**. The probability of death as a function of the number of errors on the Modified Mini-Mental State Examination Score. The dots represent empirical data. The blue line represents the model fit of the probability of death between the first (CSHA-1) and second waves (CSHA-2). The red line represents the model fit of the probability of death between the second (CSHA-2) and third (CSHA-3) waves.

The distribution of the 3MS was virtually identical over all 3 waves (Figure 1). This occurred even though loss from one wave to the next was informative: mortality (which was generally higher for the second transition than the first) was clearly related to the 3MS score (Figure 2). In each wave, survival decreased as the number of 3MS errors increased, saturating in both waves at about 50 errors (Figure 2).

**Figure 3:**
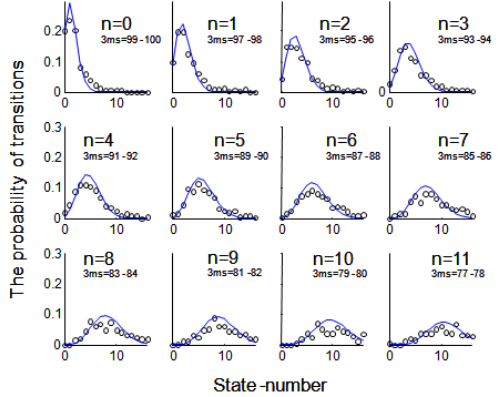
**Transitions in cognitive states from CSHA-1 to CSHA-2 as represented by errors on the Modified Mini-Mental State Examination Score**. Transitions from CSHA-1 to CSHA-2. In each panel, each cell represents the cognitive state at baseline. Each X axis represents the cognitive state at follow-up. Each Y axis represents the probability of transition to the new cognitive state. The dots represent empirical data. The solid lines represent the model fit.

**Figure 4:**
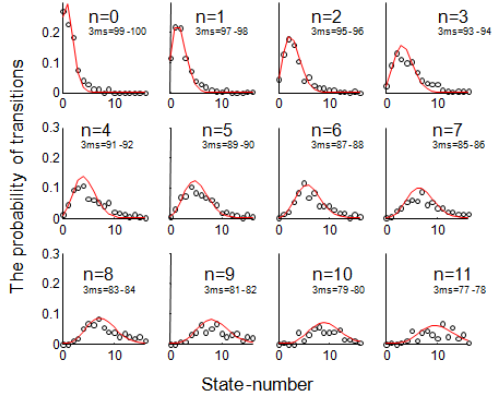
**Transitions in cognitive states from CSHA-2 to CSHA-3 as represented by errors on the Modified Mini-Mental State Examination Score**. Transitions from CSHA-2 to CSHA-3. In each panel, each cell represents the cognitive state at baseline. Each X axis represents the cognitive state at follow-up. Each Y axis represents the probability of transition to the new cognitive state. The dots represent empirical data. The solid lines represent the model fit.

In each wave, the transitions between cognitive states as represented by the 3MS errors were well fitted by the modified Poisson distribution (Table 1). The fit was very high (r>0.98) in both waves, so that the displays of the transition probabilities are virtually indistinguishable in the two waves: CSHA-1 to CSHA-2 (Figure 3) and CSHA-2 to CSHA-3 (Figure 4). The modified Poisson distribution represents the case that while both improvements (fewer 3MS errors) and declines (more 3MS errors) are possible, in general, there are more declines than improvements (Figures 3, 4). The modal transition state for *n *transitions at baseline is generally *n *+ 1 at follow-up. Consistent with the observed increase in mortality with increasing 3MS error, the area under each curve decreases as state value increases (Figures 3, 4).

**Table 1:** Estimates of the parameters, and goodness of fit, for the transitional probabilities and for death (equations (1)-(2)) in the Canadian Study of Health and Aging (CSHA).

	CSHA (wave 1–2)	CSHA (wave 2–3)
$\bar{k_{0}}$	1.17 (1.08, 1.26)	1.42 (1.32, 1.52)
*β*_1_	0.81 (0.78, 0.85)	0.86 (0.84, 0.89)
*ln(P*_*0d*_)	-1.66 (-1.71, -1.61)	-1.95 (-2.02, -1.88)
*β*_2_	0.09 (0.08, 0.09)	0.09 (0.08, 0.09)
*r*	0.99	0.98
*R*^2^	0.98	0.96

$\bar{k_{0}}$ is the mean number of cognitive errors in transition for the people who had zero errors at baseline (at the zero state) and *P*_*0d *_is the probability of 5-year mortality for the people at the zero state (with zero cognitive errors at baseline). The increments *β*_1 _and *β*_2 _(for cognitive transitions and mortality, respectively) show how these values change with the baseline error state number. Note that the background parameters $\bar{k_{0}}$, ln(P_od_) are significantly different between two transitions while the increments (*β*_1_, *β*_2_) are not.

## Discussion

We have demonstrated that changes in cognition with age are highly characteristic and can be well modeled, with very high fit, by a modified Poisson distribution. The model has four parameters that are easy to understand. Two are 'background' parameters that reflect the risk of accumulating more cognitive errors, or of dying, for a person with no errors at baseline; these can be considered the background risk. The other two reflect the incremental risk of having more than 0 errors at baseline. This modified Poisson distribution – the modification is that the parameters are state dependent – corresponds to changes in other deficits which accumulate with age [11,12,21]. In this way, these analyses contribute to a more general model about the accumulation of deficits with age

Our data must be interpreted with caution. The 3MS is not a comprehensive measure of all aspects of brain function, notably having less emphasis on executive function than do contemporary measures such as the Montréal Cognitive Assessment [22]. The CSHA, though large, has loss to follow-up at each wave for reasons other than death, although the extent of the loss is comparable to (and commonly less than) other cohort studies [23]. Cognitive aging is obviously more complex than errors on the 3MS. Even so, the data suggest that in some fundamental ways, brain aging corresponds to a more general pattern of aging seen in our earlier inquiries [11,12,21].

We were interested to observe the extent to which this model corresponds both to changes in the 3MS score and to our experience in modeling age-related deficit accumulation in general. In this way, the data potentially contribute to our understanding of how to model ageing. The robustness of the modified Poisson distribution as a general age-related deficit accumulation model is suggested by its having been replicated in the Canadian Study of Health and Aging [11], the Gothenburg H-70 cohort study [12] and the National Population Health Survey [21]. By accounting for both improvements and declines, a model such as this has the potential to clarify observations about changes in brain aging that largely have remained conceptual [24]. The model also opens up the possibility of evaluating risk factors for the background and increment parameters.

The improvements in cognitive states, across all levels of cognition, that are captured with this model have implications for the debate about the intermediate state between normal cognitive function and dementia. The intermediate state is variably described, with the terms "Mild Cognitive Impairment" (MCI) and "Cognitive Impairment, No Dementia" (CIND) predominating in North America. As reviewed elsewhere [25], a remarkable feature of clinic-based accounts of MCI, in comparison with population studies, has been the comparatively small number of people seen to have improved in clinic studies. It is not clear whether this lack of improvement in clinic-based studies is due to self-selection (although a multi-centre clinic-based study that investigated the possibility of improvement found it more often than other studies have reported [26]) or whether the supposed improvement in population studies is simply diagnostic instability. Our data go very much against diagnostic instability, as improvement seems to be a continuous feature in all cognitive states, including MCI/CIND.

Of note, whether in modeling cognitive errors specifically, or cognitive age-associated deficits in general, incremental changes in the deficit count have cumulative impacts on adverse outcomes such as death. In other words, the state of the individual – here, the cognitive state – has a quantifiable impact on outcomes. The high fit suggests that, at some level of deficit accumulation, the number of deficits is more important than exactly which ones are present. If so, this has implications for the way that we think about the impact of specific diseases in ageing, in the face of many other decrements. The relationship between a specific disease and its presentation is increasingly seen as having been undermined by disease presentation in elderly people [27], especially those who are frail. Instead, it is suggested that we should look to so-called 'geriatric syndromes' to understand illness in these people [28]. Although it might be that some of the geriatric syndromes carry a high degree of specificity in relation to deficit accumulation, this needs to be investigated, lest we turn over one orthodoxy (of disease) only to accept another (of syndromes) that might rest on just as insecure a foundation.

Of note, the introduction of separable background and incremental parameters also allows us to explore whether all risk factors that are associated with late life decrements, such as cognitive impairment, are equally associated with variability in both background and increments. These investigations have the potential to inform the modeling of ageing, perhaps by distinguished more fixed factors from more mutable ones. The model of deficit accumulation also needs to be evaluated with cellular and animal data. Such considerations are motivating additional inquiries by our group.

## Conclusion

A general model of aging as deficit accumulation described how cognitive errors accumulate with age. The model shows that the possibility of improvement is non-trivial, and needs to be considered in any general account of how cognition changes with age.

## Competing interests

The author(s) declare that they have no competing interests.

## Authors' contributions

AM & KR participated in the conceptualization, design of the study, statistical analysis, writing and revision. Both authors wrote and approved the final manuscript.

## Pre-publication history

The pre-publication history for this paper can be accessed here:

http://www.biomedcentral.com/1471-2318/8/3/prepub
